# Mild Late-Onset Sensory Neuropathy Associated with Heterozygous Missense GDAP1 Variants

**DOI:** 10.1155/2022/7492077

**Published:** 2022-05-24

**Authors:** Nivedita U. Jerath

**Affiliations:** AdventHealth Neuroscience Institute, 1573 West Fairbanks Avenue, Suite 210 Winter Park, Orlando, FL, USA

## Abstract

This study presents the clinical and electrophysiological findings of four subjects with a pathogenic heterozygous GDAP1 variant causing Charcot–Marie–Tooth disease 2K (CMT2K) and one additional subject with an uncertain GDAP1 variant and clinical findings of CMT 2K. The study evaluated these five subjects using clinical, laboratory, electrophysiological, and genetic testing. The findings showed that clinical features demonstrated no pes cavus, no significant weakness in the hands or feet, normal reflexes in four out of the five subjects, and mild to normal electrodiagnostic findings. The variant was associated with painful and numb feet with diminished sensation to pinprick. This study suggests that GDAP1 variants may be associated with very mild, predominantly sensory Charcot–Marie–Tooth disease, warranting continuing research for this type of the disease.

## 1. Introduction

Charcot–Marie–Tooth disease (CMT) reflects a group of inherited neuropathies affecting 1 in 2,500 individuals [1–3]. CMT type 1A is the most common CMT, accounting for approximately 60% of those with a genetic diagnosis [4]. Classically, CMT is characterized by distal weakness, foot structural deformities, sensory abnormalities, areflexia, and abnormalities in gait [5].

Genetic advances have allowed the classic CMT phenotype to be expanded and evaluated for idiopathic unexplained neuropathies [4]. The rare forms of CMT still need to be understood. This study aims to elucidate our understanding of the clinical features of heterozygous GDAP1 mutations.

GDAP1 mutations can present in both heterozygous and homozygous forms [2, 6]. GDAP1 mutations are responsible for demyelinating intermediate and axonal recessive CMT and CMT 2K, a rare dominant axonal CMT [5, 7–10]. The majority of GDAP1 mutations are biallelic giving rise to autosomal recessive CMT, which can present as either demyelinating [5], axonal [10], or intermediate CMT [11].

Although the homozygous presentation is typically very severe with early childhood onset with motor and sensory involvement and possible vocal fold paresis, the heterozygous form seems much less severe with a late-onset mild sensory presentation [2].

This study further describes the clinical presentation of GDAP1 mutations and adds to the knowledge of heterozygous GDAP1 mutations.

## 2. Methods

All subjects received care at the AdventHealth Orlando Neuroscience Institute. All had neurological records evaluated. In addition, four out of the five individuals had electrodiagnostic testing performed. Genetic testing for inherited neuropathies was performed through Invitae (San Francisco, CA) [12]. Three pathogenic variants in the GDAP1 gene were identified, and one variant of uncertain significance in the GDAP1 gene was also detected.

## 3. Results

**Case 1 .**

As a child, this subject had no difficulties with running, walking, roller skating, or biking. She was not athletic and was the slowest runner compared to her peers at school but was healthy and active. Her symptoms first started in her 50s when she started to have difficulty with fatigue and numbness in her toes. Occasionally, she will have pain. She has no weakness but does have some difficulty with climbing stairs. Her son was diagnosed with CMT at age 10 due to high arches, which prompted her evaluation for CMT.

**Case 2 .**

In her youth, she had no difficulties with running, walking, balance, or athletics. She was a slow runner in general and had weak ankles. Her symptoms started in her 20s when she experienced burning foot pain after standing for prolonged periods. In her 40s, she felt that she started to develop numbness in her feet and mild difficulty with dexterity in her hands. Examination revealed absent Achilles reflexes.

**Case 3 .**

As a child, she had no difficulties with running, walking, roller skating, or biking. Although she was not athletic, she was a fast walker. She had some mild tremors throughout her life. She developed burning, tingling, and extreme sensitivity to cold at age 67. She has numbness in her thumbs and paresthesias in her hands and feet. Neurological examination is intact except for absent Achilles reflexes. Both her father and her daughter have CMT 2K.

**Case 4 .**

Daughter of the Case 3 proband: as a child, she was athletic and the fastest runner compared to her peers in school. She had cramps and growing pains but no other symptoms; she has normal foot structure. Then, in her 40s, she developed numbness in her left toes and obtained genetic testing due to her mother's CMT diagnosis.

**Case 5 .**

As a child, he was athletic. In his early 50s, he started to develop constant daily pain in both plantar aspects of his feet below the middle toe area. He also has a numb feeling as if someone wrapped duct tape around his foot. Despite pain relieving interventions, including neurontin, pregabalin, marijuana, and acupuncture, he has not had pain relief. He has difficulty walking due to his balance. He had a ruptured Achilles tendon. He walks and rides his bike daily. EMG/NCS showed minor abnormalities to suggest a possible sensorimotor polyneuropathy. Exam is significant only for absent ankle jerk reflexes and pinprick sensation decreased up to ankle.

## 4. Discussion

CMT can have various symptoms and signs, including mild paresthesias, as evidenced in this study. In some cases, genetic testing can reveal underlying causes of idiopathic mild neuropathies.

The findings from the subjects with a heterozygous pathogenic GDAP1 missense variant (Table 1) demonstrate that all presented with a mild sensory neuropathy; all except one subject had a late-onset neuropathy developing after age 40. Electrodiagnostic testing was normal in one of the five individuals, with the other two individuals having minor nerve conduction abnormalities. Neurological exam was normal except for decreased pinprick in proband 1, absent reflexes in proband 2, and absent Achilles reflex in proband 3. There was no family history in proband 2. Proband 1 had a son with high arches in his feet at age 10. Proband 3 has a daughter with numbness in her left toe and a father who had a neuropathy. Proband 5 developed pain in his feet in his 50s.

Previous studies of heterozygous GDAP1 variants suggest a late-onset, slowly progressive, and mild neuropathy [7, 9]. Another earlier study suggested a wide variability in age of onset and severity [13]. Zimon et al. (2011) also reported heterozygous GDAP1 variants with various age onset, but unlike our study, some subjects presented with walking difficulties. However, similar to our study, some subjects were asymptomatic, perhaps due to incomplete penetrance or the nature of the heterozygous form. Electrodiagnostic studies revealed an axonal or an intermediate pattern. A previous study suggested that five variants in the GDAP1 gene (p.Arg120Gly, p.Arg120Trp, p.His123Arg, p.Gln218Glu, and p.Arg226Ser) had a milder and indolent clinical course [14]. In general, heterozygous carriers were milder.

The GDAP1 variant is a rare CMT variant in which autosomal recessive and dominant forms can be seen [2]. The GDAP1 gene in a heterozygous form results in a suspected pathology localized to abnormalities in mitochondrial fusion [15].

Classical CMT2 is an axonal CMT with reduced amplitudes [16]. Our subjects had completely normal or borderline electrodiagnostic testing at the time of their evaluations, as seen previously [6]. This can make the diagnosis challenging.

Our subjects were comprised of four women and one man. Men may need to be separately investigated as there might be a gender difference or variability among this population [2].

In summary, CMT should be considered a possible differential in mild late-onset sensory neuropathies with normal to borderline abnormal nerve conduction studies.

## Figures and Tables

**Table 1:** Clinical Results of individuals with a heterozygous GDAP1 missense variant.

Proband	Variants found in heterozygous state	Onset	Clinical symptoms	EMG/NCS	Family history
1	GDAP1 c.358C > T (p.Arg120Trp), classified as pathogenic	20s	Burning feet, intermittent hand numbness; decreased pinprick to midcalf	Absent sural sensory responses bilaterally	Son with high arches at age 10
2	GDAP1 c.358C > T (p.Arg120Trp), classified as pathogenic	Early 50s	Not athletic as a child, numb toes, reflexes absent	Right ulnar sensory response mildly reduced amplitude	None
3	GDAP1 c.811G > A (p.Gly271Arg), classified as pathogenic	Late 60s	Paresthesias and numbness in thumbs and toes, absent achilles reflexes	Normal	Father with neuropathy
4 (proband 3's daughter)	GDAP1 c.811G > A (p.Gly271Arg), classified as pathogenic	40s	Fast runner as a child but had cramps; numb toes on left foot	Not performed	
5	GDAP 1 c.1006G > T (p.Ala336Ser); classified as a variant of uncertain significance.	50s	Was able to run, walk, and bike as a child up until his 50s when he developed pain in both feet as if “duct tape” around it.	Left superficial peroneal and ulnar sensory and left tibial motor response mildly reduced amplitude	Mother with neuropathic pain.

## Data Availability

The data used to support this article can be made available by the corresponding author on request.
